# Supplementary material for: Molecular determinants of substrate specificity in the efflux pump CraA from Acinetobacter baumannii
Source: Microbiol Spectr. 2025 Jul 7;13(8):e01119-25. doi: 10.1128/spectrum.01119-25 (PMC12323617; doi:10.1128/spectrum.01119-25)
Supplement: Supplemental material — Tables S1 to S3; Fig. S1 to S12. [file spectrum.01119-25-s0002.pdf]

Molecular determinants of substrate specificity in *Acinetobacter baumannii* multidrug efflux pump CraA

Wuen Ee FOONG[1,2,3], Xinxin XIANG[1], Klaas M. POS[3,*], Heng-Keat TAM[1,2,3,4,*]

[1]Department of Biochemistry and Molecular Biology, Hengyang Medical School, University of South China, Hengyang, Hunan 421001, China.

[2]Department of Medical Microbiology, Hunan Provincial Key Laboratory for Special Pathogens Prevention and Control, Hengyang Medical School, University of South China, Hengyang, Hunan 421001, China.

[3]Institute of Biochemistry, Goethe-University Frankfurt, Max-von-Laue-Str. 9, D-60438 Frankfurt am Main, Germany.

[4]National Health Commission Key Laboratory of Birth Defect Research and Prevention, Hunan Provincial Maternal and Child Health Care Hospital, Changsha, Hunan 410008, China.

*Corresponding author. Tel: +49-(0)69-798 29251; Fax: +49-(0)69-798 29201; E-mail: pos@em.uni-frankfurt.de; tamhk60@hotmail.com

TABLE S1 Primer sequences. The highlighted nucleotides represent the mutated residues.

**Cloning via Gibson assembly**	
**Plasmids**	**Primer sequence (5'-3')**
pTTQ18_CraA_WT	pTTQ18-F CATCACCATCATCATCATCATTAATAA<br>pTTQ18-R CGCTGTTTCCTGTGTGAAATTGTTATCCGCTC<br>CraA-F ATTTCACACAGGAAACAGCGATGCTGAGACATAGTGGCCGCT<br>CraA-R ATTAATGATGATGATGATGGTGATGATGTAAGTCTTGTGCAACTCTTTC
pBAV1K_CraA	pBAV1K-F GGCCCGATCGATGCCGCCGCTTAATTAA<br>pBAV1K-R CTGAGGCCTGCAGCGGCCGCGAA<br>CraA_comp-F CGCGGCCGCTGCAGGCCTCAGGCTGATTCTGAGTGCTTGTACACC<br>CraA_comp-R GCGGCGGCATCGATCGGGCCTTAATGTAAGTCTTGTGCAACTC
**Cloning with restriction enzymes**	
pBIISK_CraA_updown	CraA_up_NotI-F ATTAGCGGCCGCGCTTCCACATAACTATCTACATCAG<br>CraA_up_BamHI-R ATTAGGATCCACGTCGTACGGTTCAACGCTGTCG<br>CraA_down_BamHI-F ATTAGGATCCCAGGAAAGAGTTGCACAAGACTTAC<br>CraA_down_PstI-R ATTACTGCAGAGTTGCACAGAGCCTTAACCAAATAC<br>CraA_ctr-F CCGGATTAGGCTGCTGCAAATAGTG<br>CraA_ctr-R CTAAAGCACGGTTATTCGACCCAG
**Site directed mutagenesis**	
**Plasmids**	**Primer sequence (5'-3')**
pTTQ18_CraA_E38A	E38A-F TGCATTTGCGGTTTATATTGGTAA<br>E38A-R AATAGCACTAAAGCTAAAGGAAAC
pTTQ18_CraA_E38H	E38H-F TCACTTTGCGGTTTATATTGGTAA<br>E38A-R AATAGCACTAAAGCTAAAGGAAAC
pTTQ18_CraA_E38Q	E38Q-F TCAGTTTGCGGTTTATATTGGTAA<br>E38A-R AATAGCACTAAAGCTAAAGGAAAC
pTTQ18_CraA_E38D	E38D-F TGACTTTGCGGTTTATATTGGTAA<br>E38A-R AATAGCACTAAAGCTAAAGGAAAC
pTTQ18_CraA_D46A	D46A-F GGTAATGCCCTGATTCAGCC<br>D46A-R AATATAAACCGCAAATTCAAATAGC
pTTQ18_CraA_D46H	D46H-F ATCACCTGATTCAGCCAGC<br>D46H-R TACCAATATAAACCGCAAATTC
pTTQ18_CraA_D46N	D46N-F ATAACCTGATTCAGCCAGC<br>D46H-R TACCAATATAAACCGCAAATTC
pTTQ18_CraA_D46E	D46E-F ATGAACTGATTCAGCCAGC<br>D46H-R TACCAATATAAACCGCAAATTC
pTTQ18_CraA_Y42A	Y42A-F GTTGCTATTGGTAATGACCTG<br>Y42A-R CGCAAATTCAAATAGCACTAAAGC
pTTQ18_CraA_Y42F	Y42F-F GTTTTTATTGGTAATGACCTG<br>Y42A-R CGCAAATTCAAATAGCACTAAAGC
pTTQ18_CraA_Y42L	Y42F-F GTTTTAATTGGTAATGACCTG<br>Y42A-R CGCAAATTCAAATAGCACTAAAGC
pTTQ18_CraA_N45A	N45A-F GCAGACCTGATTCAGCCAGC<br>N45A-R ACCAATATAAACCGCAAATTCAAA
pTTQ18_CraA_M70A	M70A-F CGCCGTCTTCAGCGTCATTT

	M70A-R CCCAAGTTGCACTTACACCGAAAT
pTTQ18_CraA_Y73A	Y73A-F TGCCTTATTAGGTGGTGCATC<br>Y73A-R AATGACATTGAAGACGGCGC
pTTQ18_CraA_Y73F	Y73F-F TTCTTATTAGGTGGTGCATCTG<br>Y73F-R AAATGACATTGAAGACGGCGC
pTTQ18_CraA_Y73L	Y73L-F CTGTTATTAGGTGGTGCATCTG<br>Y73F-R AAATGACATTGAAGACGGCGC
pTTQ18_CraA_L74A	L74A-F GTCATTTTATGCATTAGGTGGTG<br>L74A-R ATTGAAGACGGCGCCCAAGTT
pTTQ18_CraA_R124A	R124A-F CTGACATTAGCTTTTTTACAAGG<br>R124A-R AAAGTGTTCAATTTGTCGCGTTA
pTTQ18_CraA_R124H	R124H-F CTGACATTACATTTTTTACAAGG<br>R124A-R AAAGTGTTCAATTTGTCGCGTTA
pTTQ18_CraA_L131A	L131A-F GGCAAGCGTAATTTCAGCAG<br>L131A-R CCAATACCTTGTAAAAAACGT
pTTQ18_CraA_S135A	S135A-F TTGCAGCAGTGGGATATGCC<br>S135A-R TTACGCTTAACCCAATACCTTG
pTTQ18_CraA_Y139A	Y139A-F GGAGCTGCCGCAATTCAGGAA<br>Y139A-R CACTGCTGAAATTACGCTTAAC
pTTQ18_CraA_M158A	M158A-F CCTCGCGGCAAATATTTCATTGC<br>M158A-R GCCATGACTTTAATCGCATCACG
pTTQ18_CraA_S162A	S162A-F ATTGCATTGCTTGCGCCTTTGC<br>S162A-R ATTTGCCATGAGGGCCATGAC
pTTQ18_CraA_L163A	L163A-F ATTTCAGCGCTTGCGCCTTTG<br>S162A-R ATTTGCCATGAGGGCCATGAC
pTTQ18_CraA_P166A	P166A-F ATTGCTTGCGGCTTTGCTAG<br>P166A-R GAAATATTTGCCATGAGGGCC
pTTQ18_CraA_L246A	L246A-F GTATGCCGGCTATGCTTTGG<br>L246A-R CCACAAGTGGTAAGGCCAAC
pTTQ18_CraA_M247A	M247A-F GTATGCCGCTTGCGCTTTGG<br>L246A-R CCACAAGTGGTAAGGCCAAC
pTTQ18_CraA_I250A	I250A-F GGGCTGCATTATCGCCAATTATT<br>I250A-R AAAGCATAAGCGGCATACCCAC
pTTQ18_CraA_Y268A	Y268A-F AAGCTGGTTTAGCACAGTTCC<br>Y268A-R GCACACTCGTTAGCTTCAGT
pTTQ18_CraA_F276A	F276A-F CCGGTAGCTTTAGGTTTAATTGT<br>F276A-R GAACTGTGCTAAACCATATTGCA
pTTQ18_CraA_L279A	L279A-F TTAGGTGCAATTGTTGGTAACAT<br>L279A-R AAATACCGGGAACTGTGCTA
pTTQ18_CraA_N283A	N283A-F GTTTAATTGTTGGTGCCATTGTTT<br>N283A-R CTAAAAATACCGGGAACTGTGC
pTTQ18_CraA_E338A	E338A-F TTGGTGCAGGAATTAGCTTCTC<br>E338A-R AACAAATGAGTGTCATACCTATTAA
pTTQ18_CraA_E338D	E338D-F GATGGAATTAGCTTCTCAGTGTTG<br>E338D-R ACCAAAACAAATGAGTGTCATAC

pTTQ18_CraA_E338I	E338I-F ATTGGAATTAGCTTCTCAGTGTTG<br>E338D-R ACCAAAACAAATGAGTGTCATAC
pTTQ18_CraA_E338N	E338N-F AACGGAATTAGCTTCTCAGTGTTG<br>E338D-R ACCAAAACAAATGAGTGTCATAC
pTTQ18_CraA_E338Q	E338Q-F CAGGGAATTAGCTTCTCAGTGTTG<br>E338D-R ACCAAAACAAATGAGTGTCATAC
pTTQ18_CraA_F342A	F342A-F GAATTAGCGCCTCAGTGTTGTAC<br>F342A-R CTTCACCAAAACAAATGAGTGTCATA
pTTQ18_CraA_Y346A	Y346A-F GCTCGTTTTGCGCTCATGTC<br>Y346A-R CAACACTGAGAAGCTAATTCC
pTTQ18_CraA_R347A	R347A-F GTTGTACGCTTTTGCGCTCATG<br>R347A-R ACTGAGAAGCTAATTCCTTCACC
pTTQ18_CraA_K357A	K357A-F TGAAGTGTCAGCAGGAACTGTAG<br>K357A-R GATGACATGAGCGCAAAACGGTA
pTTQ18_CraA_L368A	L368A-F GCGATGACGAGCTTCTTTGC<br>L368A-R TAGCATTGAAACAGCAGCAGC
pTTQ18_CraA_M369A	M369A-F TTGGCGACGAGCTTCTTTGC<br>L368A-R TAGCATTGAAACAGCAGCAGC
pTTQ18_CraA_F372A	F372A-F GCGCCTTTGCAATGATTG<br>F372A-R TCGTCATCAATAGCATTGAAAC
**Reverse-transcription qPCR**	
craA	Forward ATGCTCACTGGTACGCTAAT<br>Reverse GACACTTCAGATGACATGAG
rpoB	Forward GAGTCTAATGGCGGTGGTTC<br>Reverse ATTGCTTCATCTGCTGGTTG

27

TABLE S2 Inhibition of ethidium efflux of CraA wildtype and variants by CCCP. The log of the time required for the cells to remove half of the ethidium, parameter *H*, of each CraA variants, was calculated by fitting the curves of ethidium efflux to a sigmoid function as described in Materials and Methods. $t_{efflux\text{-}50\%}$, the time needed to reach a relative fluorescence of 0.5 is calculated by fitting the estimated *H* to the exponentiation function, $f(H) = e^H$. Statistical comparisons between the untreated cells (addition of DMSO) and cells treated with CCCP (dissolved in DMSO) were performed using Tukey's multiple comparison test, *** represents $p < 0.001$. All CraA variants were expressed equally well compared to wild-type CraA (Fig. S3B).

	Ethidium efflux	
**Variants**	***H***	$t_{efflux\text{-}50\%}$
WT + DMSO	4.69 ± 0.13	108.85
WT + CCCP	5.94 ± 0.13 ***	379.93
Vector + DMSO	7.43 ± 0.13	1685.81
Vector + CCCP	7.62 ± 0.13	2038.56
D46A + DMSO	6.20 ± 0.13	492.75
D46A + CCCP	6.59 ± 0.13	727.78

TABLE S3 pK_a values of membrane-embedded carboxylated residues lining the binding pocket of CraA and MdfA (PDB: 4ZOW, 1) as predicted by PROPKA3.2 (2).

**Residues**	**Ligand bound**	**Without ligand**
MdfA + chloramphenicol (PDB: 4ZOW)		
E26	8.17	7.86
D34	7.36	7.52
Initial CraA homology model		
E38	-	7.52
D46	-	6.94
E338	-	8.31
Docking pose of CraA + chloramphenicol		
E38	7.41	7.13
D46	7.94	7.48
E338	8.06	8.48
Docking pose of CraA + ethidium		
E38	7.56	7.19
D46	7.17	7.56
E338	7.77	7.61
Docking pose of CraA + mitomycin C		
E38	6.87	7.04
D46	7.06	7.58
E338	7.09	7.65
Docking pose of CraA + norfloxacin		
E38	7.84	7.53
D46	7.54	7.51
E338	6.07	6.44

FIG S1 Protein sequence alignment of CraA of *A. baumannii* AYE, (Genbank accession number: CAJ77876) and MdfA of *E. coli*. Residues lining along the binding pocket are indicated in bold. Color coded for amino acids are shown as: hydrophobic residues = red; negatively charged residues = cyan; hydrophilic residues or proline or glycine = green; positively charged residues = magenta.

```
                  10        20        30        40        50        60        70        80        90
MdfA      --------------MQNKLASGARLGRQALLFPLCLVLYEFSTYIGNDMIQPGMLAVVEQYQAGIDWVPTSMTAYLAGGMFLQWLLGPLSDR
CraA      MLRHSGRYFEVYKLMKNIQTTA--LNRTTLMFPLALVLFEFAVYIGNDLIQPAMLAITEDFGVSATWAPSSMSFYLLGGASVAWLLGPLSDR

                 100       110       120       130       140       150       160       170       180
MdfA      IGRRPVMLAGVVWFIVTCLAILLAQNIEQFTLLRFLQGISLCFIGAVGYAAIQESFEEAVCIKITALMANVALIAPLLGPLVGAAWIHVL
CraA      LGRKKVLLSGVLFFALCCFLILLTRQIEHFLTLRFLQGIGLSVISAVGYAAIQENFAERDAIKVMALMANISLLAPLLGPVLGAFLIDYV

                 190       200       210       220       230       240       250       260       270
MdfA      PWEGMFVLFAALAAVSFFGLQRAMPETATRIGEKLSLKELGRDYKLVLKNGRFVAGALALGFVSLPLLAWIAQSPIIIITGEQLSSYEYGL
CraA      SWHWGFVAIALLALLSWVGLKKQMPSHKVSVTKQ-PFSYLFDDFKKVFSNRQFLGLTLALPLVGMPLMLWIALSPIILVDELKLTSVQYGL

                 280       290       300       310       320       330       340       350       360
MdfA      LQVPIFGALIAGNLLLARLTSRRTVRSLIIMGGWPIMI-GLLVAAAATVISSHAYLWMTAGLSIYAFGIGLANAGLVRLTLFASDMSKGTV
CraA      AQFPVFLGLIVGNIVLIKIIDRLALGKTVLI-GLPIMLTGTLILILGVVWQAYLIPCLLIGMTLICFGEGISFSVLYRFALMSSEVSKGTV

                 370       380       390       400       410       420
MdfA      SAAMGMLQMLIFTVGIEISKHAWLNGGNGLFNLFNLVNGILW-LSLMVIFLKDKQMGNSHEG-
CraA      AAAVSMLLMTSFFAMIELVRYLYTQFHLWAFVLSAFAFIALWFTQPRLALKREMQERVAQDLH
```

FIG S2 Chemical structures of drugs tested in this study.

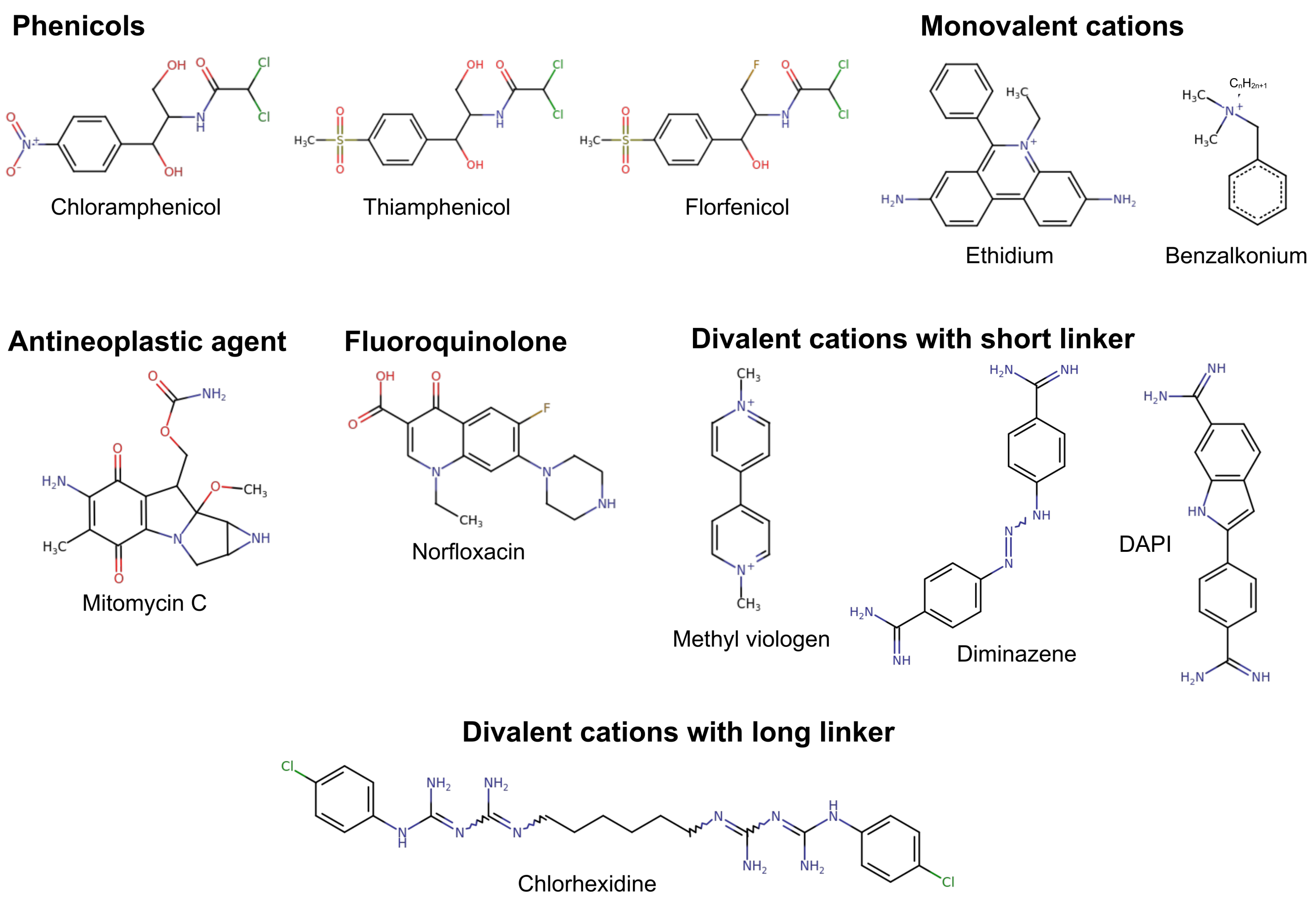

FIG S3 Characterization of wildtype CraA and its mutants. **(A)** Drug susceptibility assays of *E. coli* BW25113 Δ*emrE*Δ*mdfA* harbouring pTTQ18 empty vector, pTTQ18_craA, and CraA variants. Serially diluted cells were spotted on LB agar plates supplemented with 0.2 mM IPTG, 100 mg/L ampicillin and selected drugs (drugs and concentration given above the plate figures). Experiments were conducted at least three times and the result shown is representative. (B) The Western blot analysis of the whole cell extracts of wildtype CraA and its variants confirms that all CraA variants expressed equally well compared to wild-type CraA. The presence of two protein bands detected on the Western blot analysis is the presence of two populations of proteins, the folded and partly misfolded proteins (3).

(A)

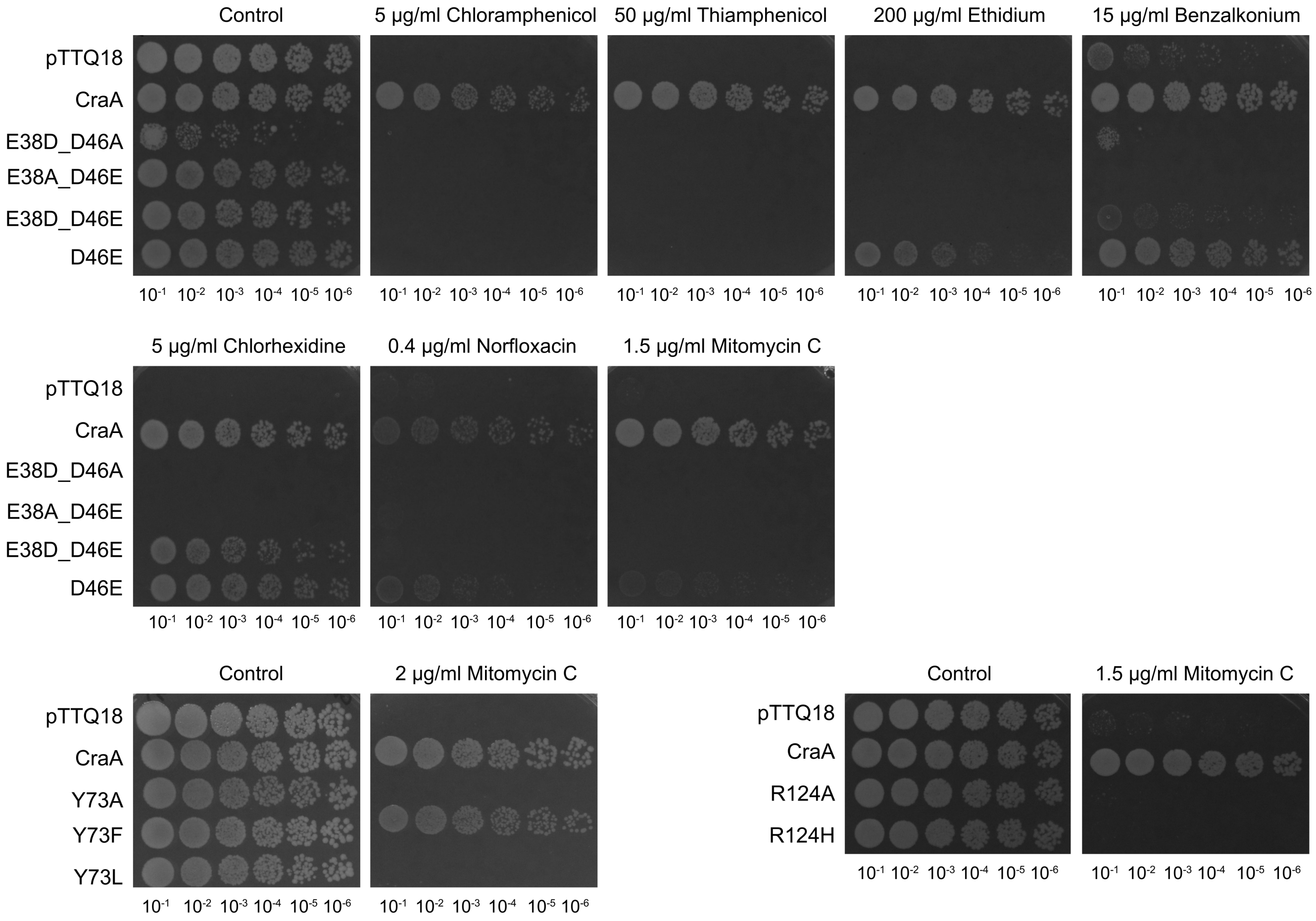

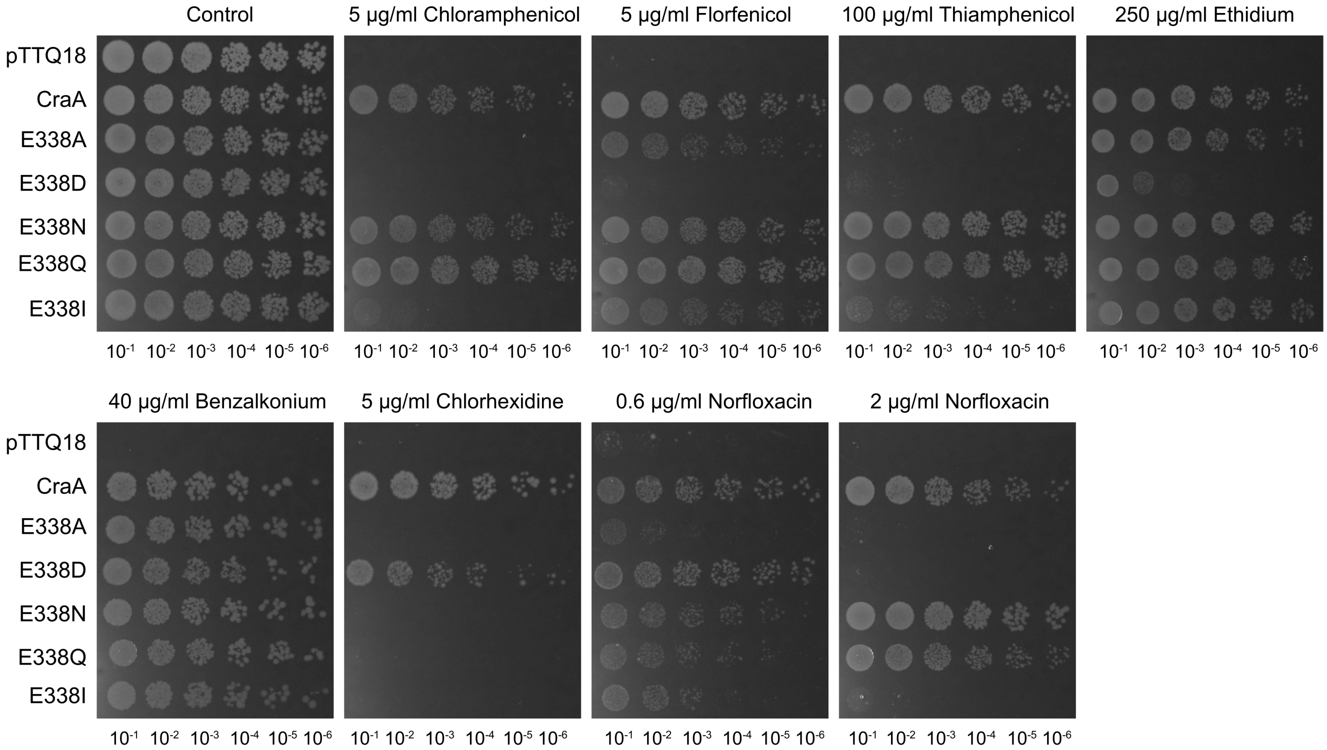
Control
5 µg/ml Chloramphenicol
5 µg/ml Florfenicol
100 µg/ml Thiamphenicol
250 µg/ml Ethidium
40 µg/ml Benzalkonium
5 µg/ml Chlorhexidine
0.6 µg/ml Norfloxacin
2 µg/ml Norfloxacin
pTTQ18
CraA
E338A
E338D
E338N
E338Q
E338I
10-1 10-2 10-3 10-4 10-5 10-6

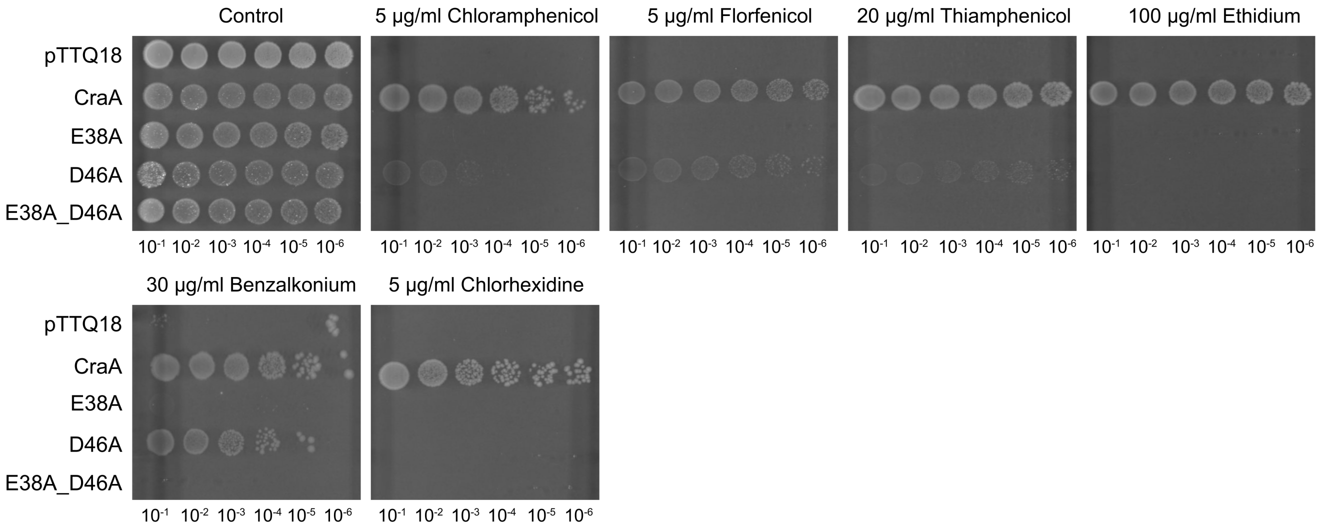
Control
5 µg/ml Chloramphenicol
5 µg/ml Florfenicol
20 µg/ml Thiamphenicol
100 µg/ml Ethidium
30 µg/ml Benzalkonium
5 µg/ml Chlorhexidine
pTTQ18
CraA
E38A
D46A
E38A_D46A
10-1 10-2 10-3 10-4 10-5 10-6

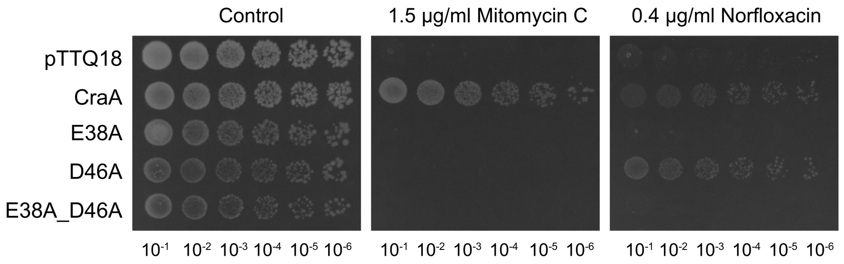
Control
1.5 µg/ml Mitomycin C
0.4 µg/ml Norfloxacin
pTTQ18
CraA
E38A
D46A
E38A_D46A
10-1 10-2 10-3 10-4 10-5 10-6

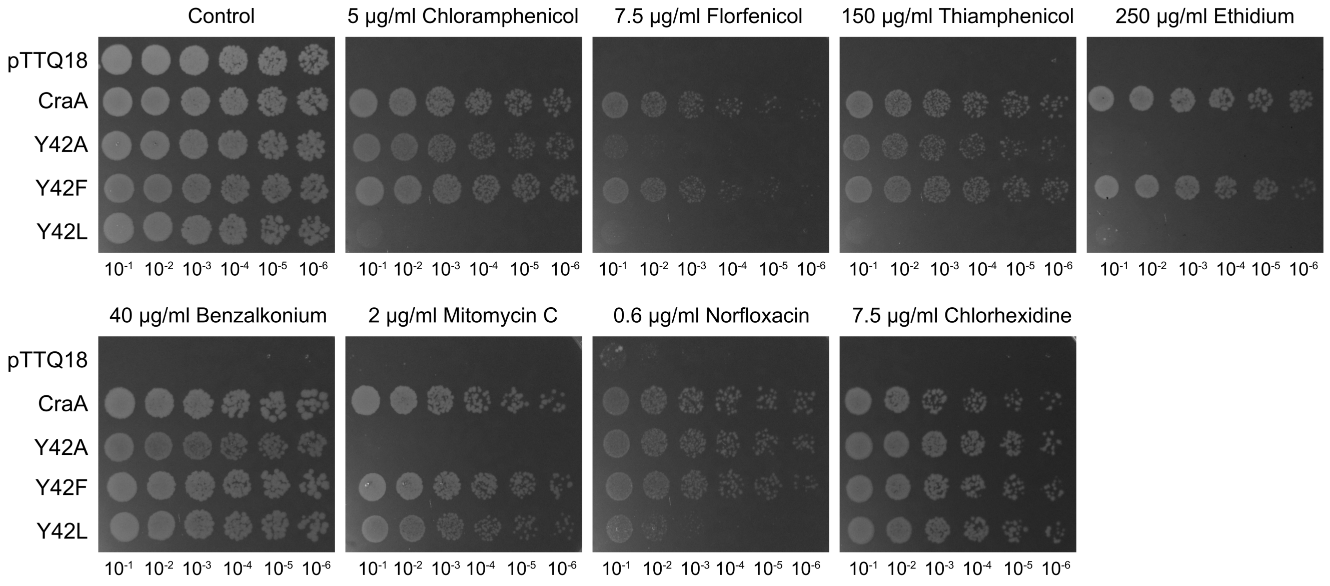
Control
5 μg/ml Chloramphenicol
7.5 μg/ml Florfenicol
150 μg/ml Thiamphenicol
250 μg/ml Ethidium
40 μg/ml Benzalkonium
2 μg/ml Mitomycin C
0.6 μg/ml Norfloxacin
7.5 μg/ml Chlorhexidine
pTTQ18
CraA
Y42A
Y42F
Y42L
10^{-1} 10^{-2} 10^{-3} 10^{-4} 10^{-5} 10^{-6}

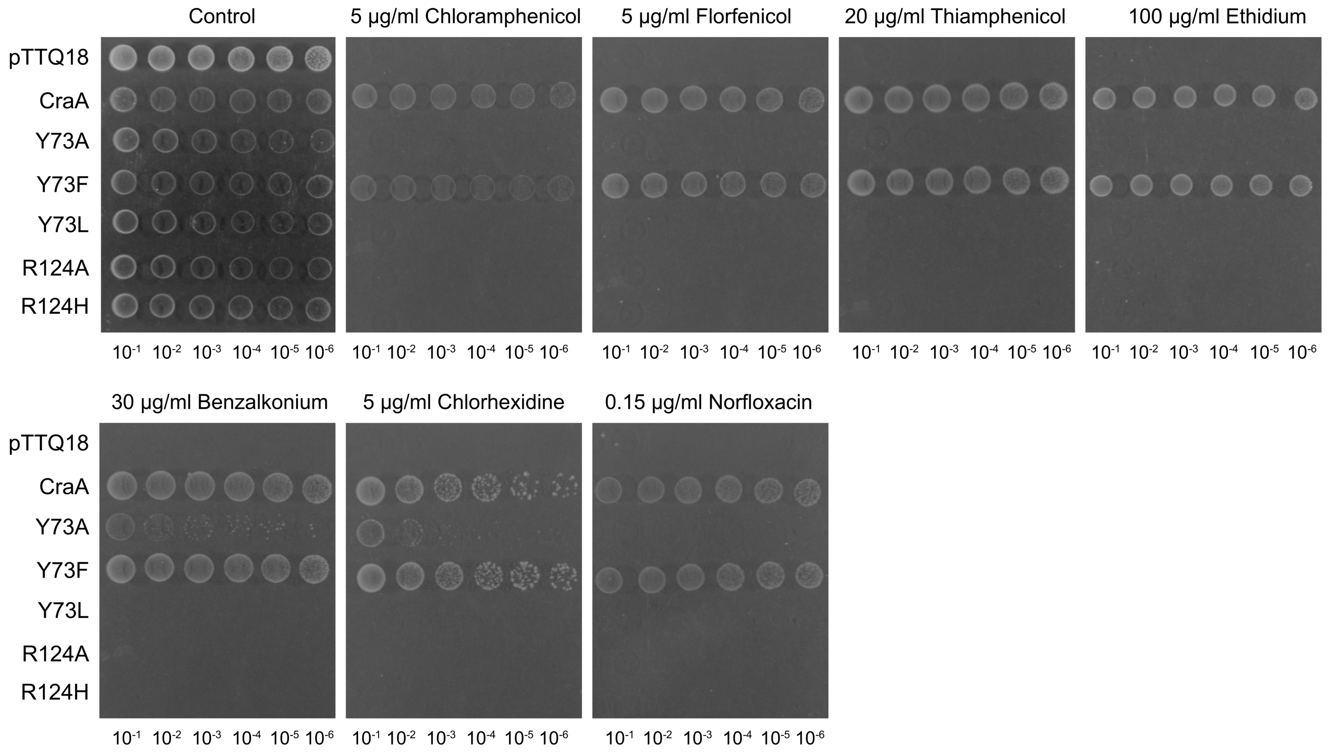
Control
5 μg/ml Chloramphenicol
5 μg/ml Florfenicol
20 μg/ml Thiamphenicol
100 μg/ml Ethidium
30 μg/ml Benzalkonium
5 μg/ml Chlorhexidine
0.15 μg/ml Norfloxacin
pTTQ18
CraA
Y73A
Y73F
Y73L
R124A
R124H
10^{-1} 10^{-2} 10^{-3} 10^{-4} 10^{-5} 10^{-6}

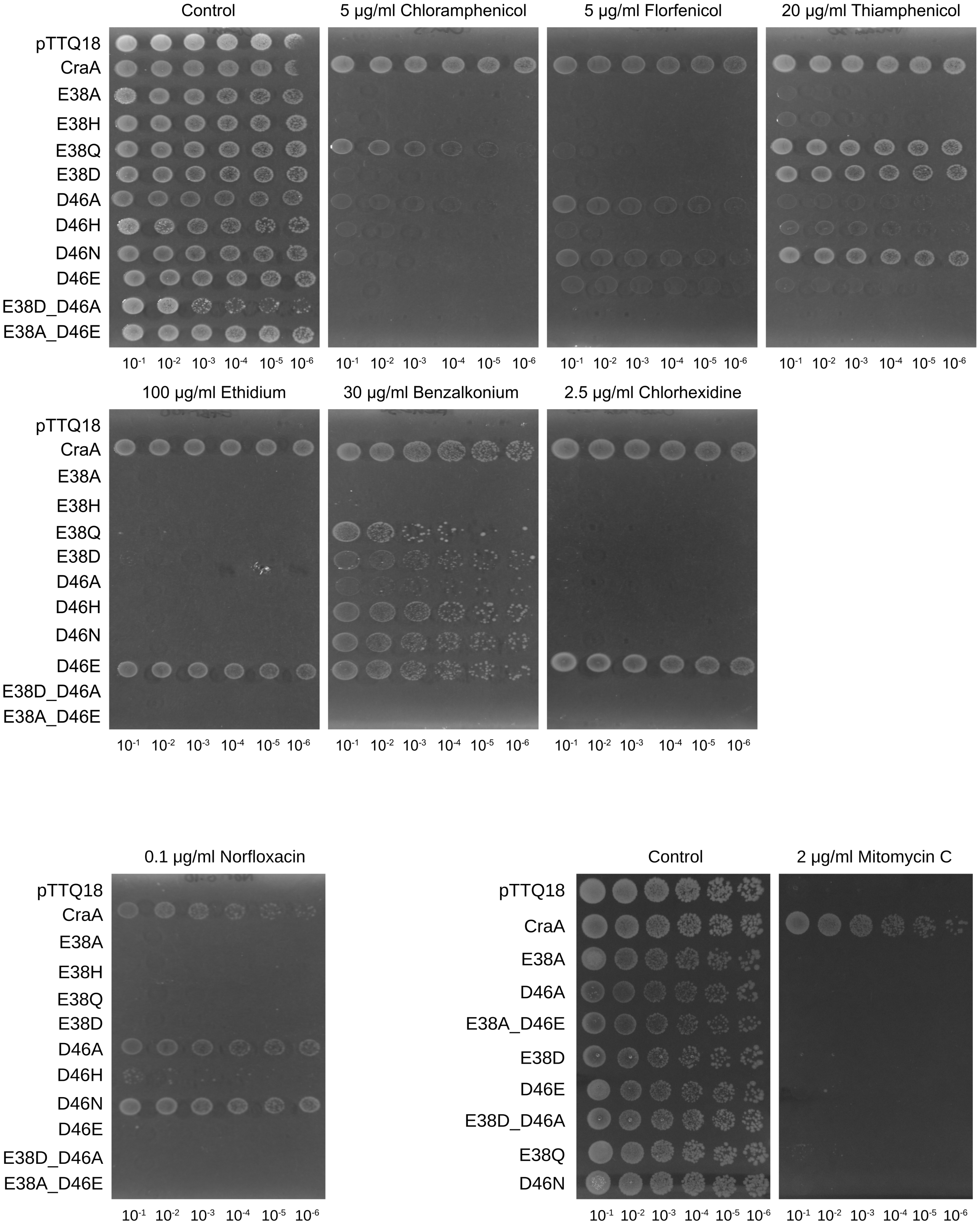
Control
5 µg/ml Chloramphenicol
5 µg/ml Florfenicol
20 µg/ml Thiamphenicol
pTTQ18
CraA
E38A
E38H
E38Q
E38D
D46A
D46H
D46N
D46E
E38D_D46A
E38A_D46E
10-1 10-2 10-3 10-4 10-5 10-6
100 µg/ml Ethidium
30 µg/ml Benzalkonium
2.5 µg/ml Chlorhexidine
0.1 µg/ml Norfloxacin
Control
2 µg/ml Mitomycin C

66

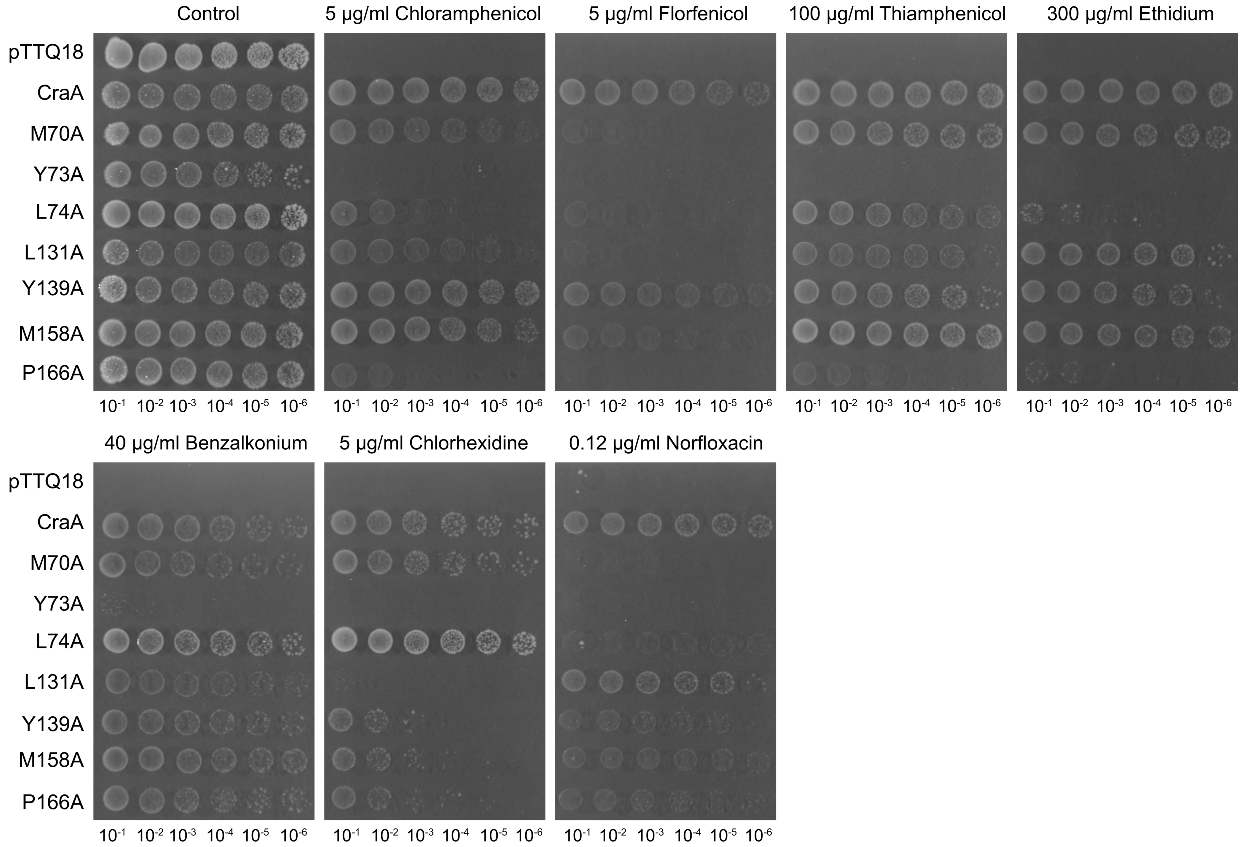
Control
5 µg/ml Chloramphenicol
5 µg/ml Florfenicol
100 µg/ml Thiamphenicol
300 µg/ml Ethidium
40 µg/ml Benzalkonium
5 µg/ml Chlorhexidine
0.12 µg/ml Norfloxacin
pTTQ18
CraA
M70A
Y73A
L74A
L131A
Y139A
M158A
P166A
10^{-1} 10^{-2} 10^{-3} 10^{-4} 10^{-5} 10^{-6}

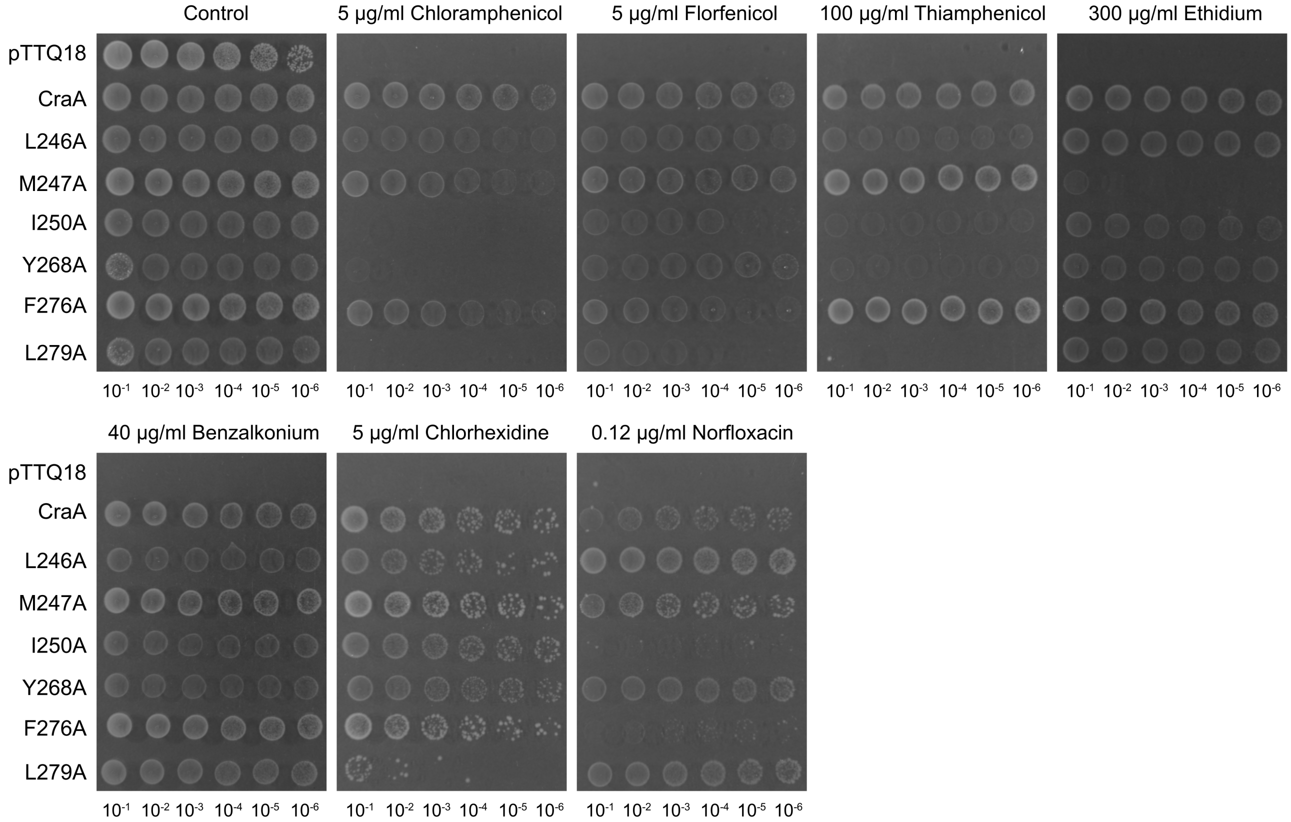
Control
5 µg/ml Chloramphenicol
5 µg/ml Florfenicol
100 µg/ml Thiamphenicol
300 µg/ml Ethidium
40 µg/ml Benzalkonium
5 µg/ml Chlorhexidine
0.12 µg/ml Norfloxacin
pTTQ18
CraA
L246A
M247A
I250A
Y268A
F276A
L279A
10^{-1} 10^{-2} 10^{-3} 10^{-4} 10^{-5} 10^{-6}

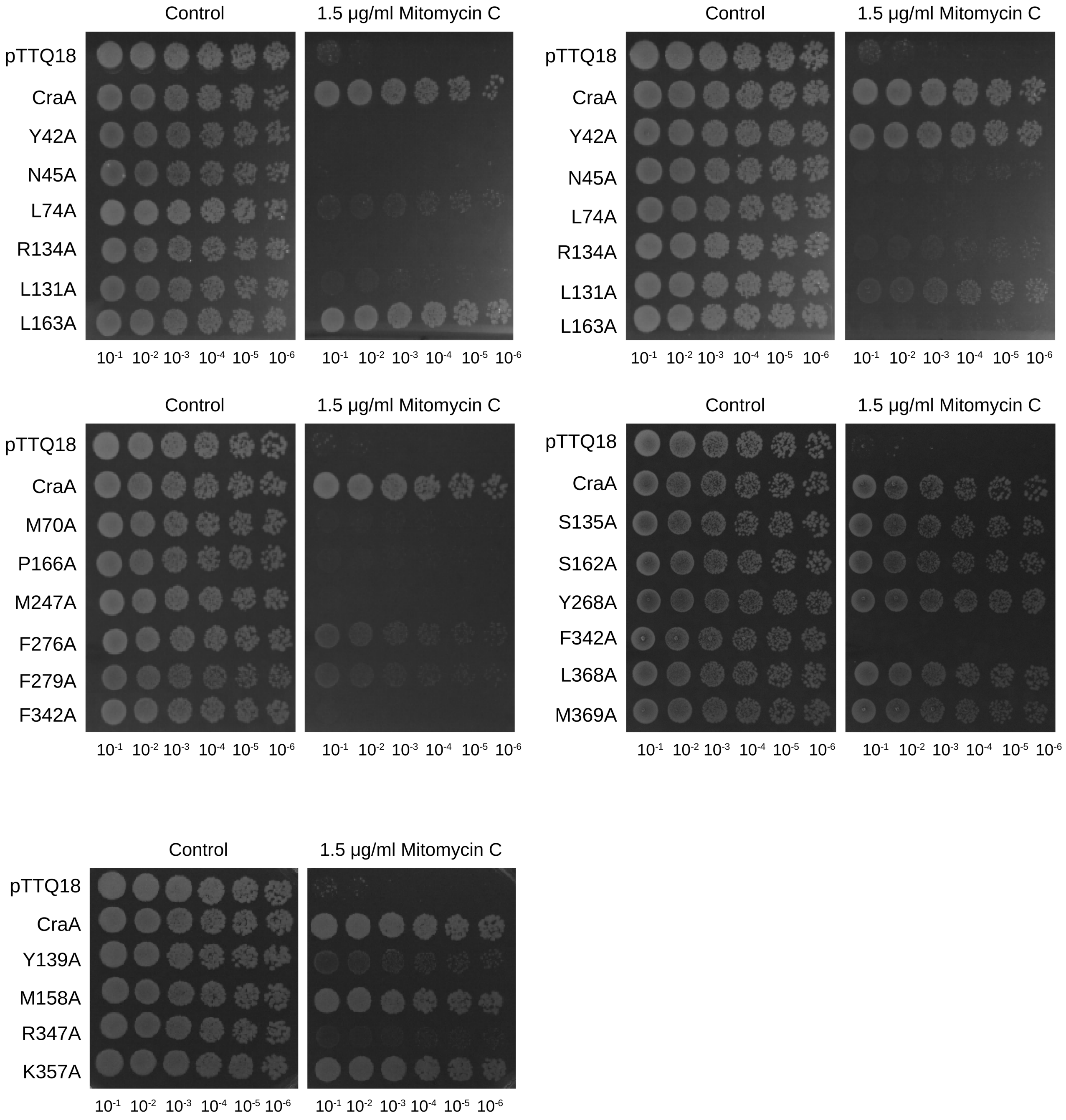
Control
1.5 µg/ml Mitomycin C
pTTQ18
CraA
Y42A
N45A
L74A
R134A
L131A
L163A
M70A
P166A
M247A
F276A
F279A
F342A
S135A
S162A
Y268A
L368A
M369A
Y139A
M158A
R347A
K357A
10-1 10-2 10-3 10-4 10-5 10-6

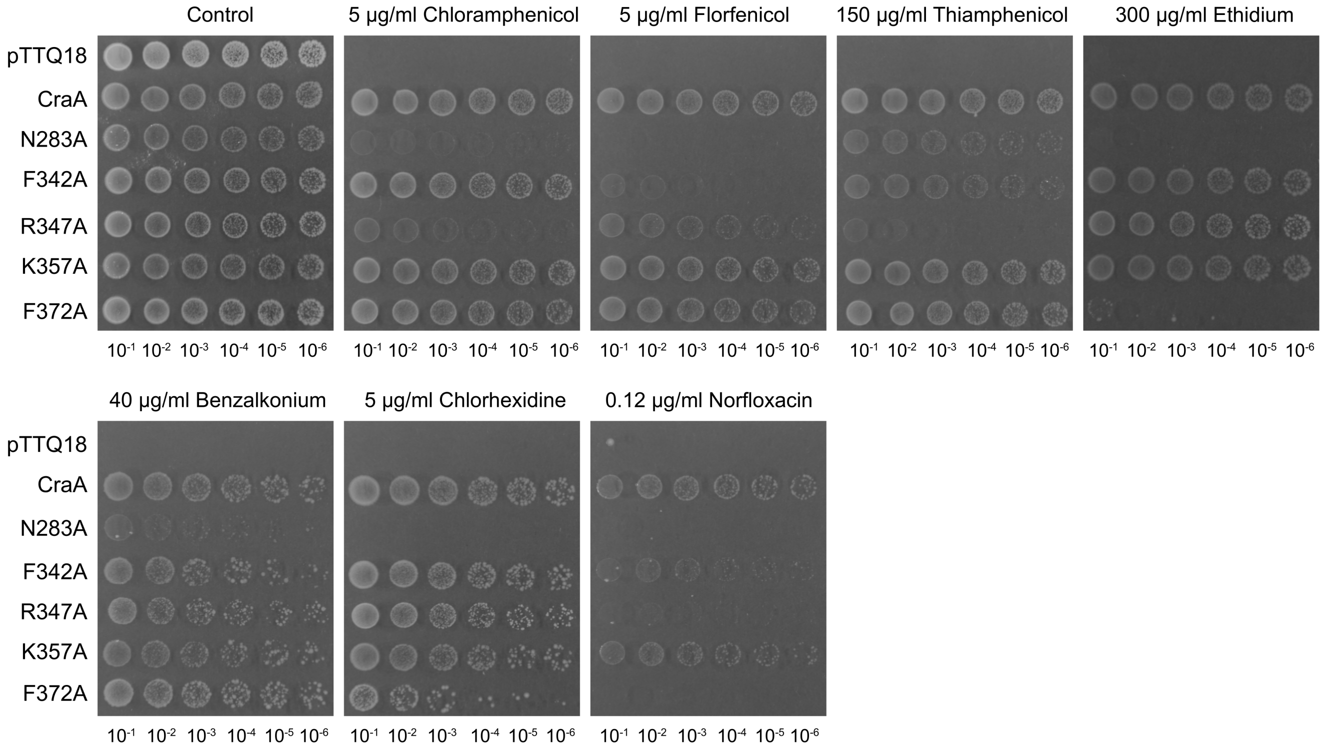
Control
5 μg/ml Chloramphenicol
5 μg/ml Florfenicol
150 μg/ml Thiamphenicol
300 μg/ml Ethidium
40 μg/ml Benzalkonium
5 μg/ml Chlorhexidine
0.12 μg/ml Norfloxacin
pTTQ18
CraA
N283A
F342A
R347A
K357A
F372A
10^{-1} 10^{-2} 10^{-3} 10^{-4} 10^{-5} 10^{-6}

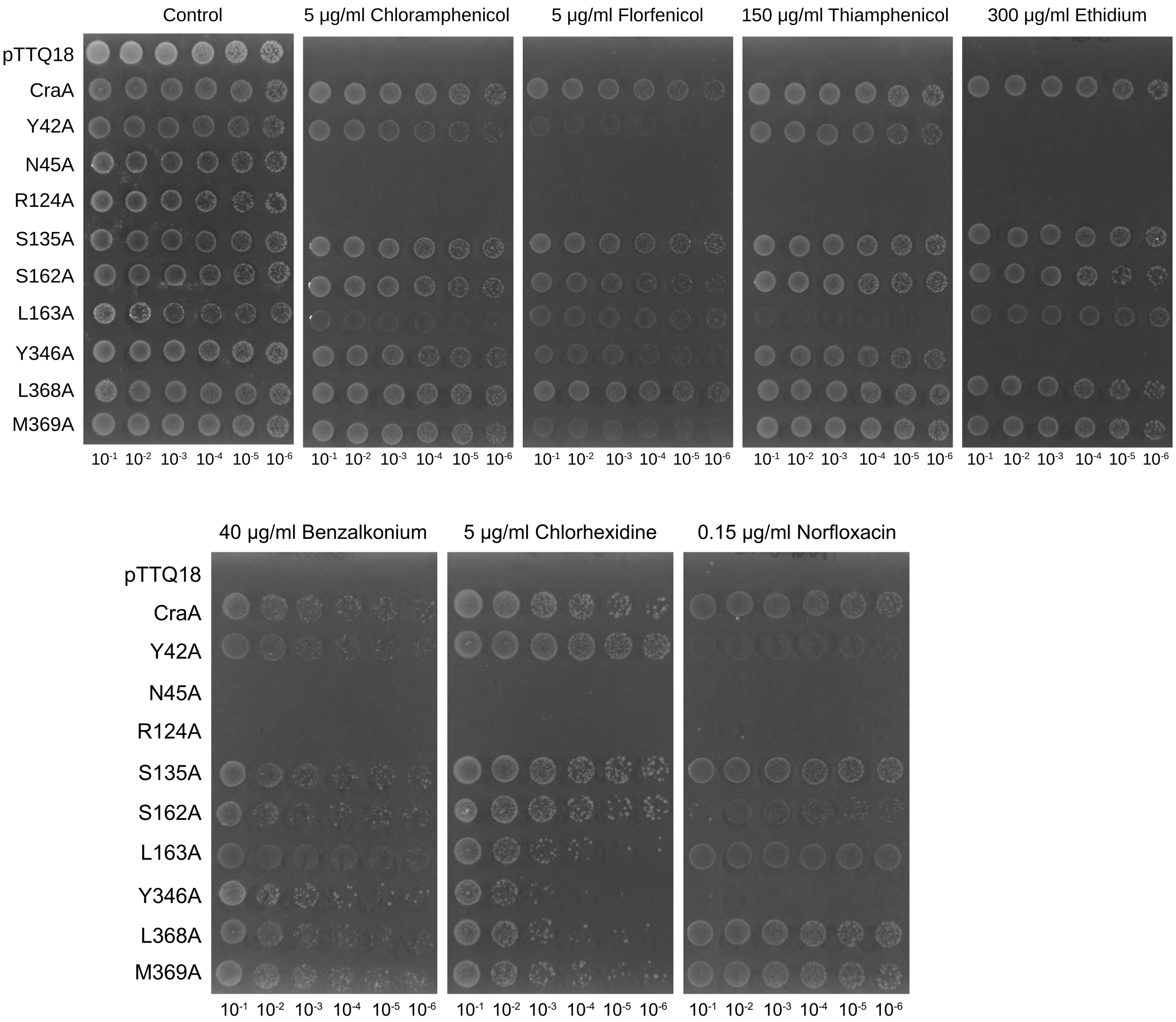
Control
5 µg/ml Chloramphenicol
5 µg/ml Florfenicol
150 µg/ml Thiamphenicol
300 µg/ml Ethidium
pTTQ18
CraA
Y42A
N45A
R124A
S135A
S162A
L163A
Y346A
L368A
M369A
10^{-1} 10^{-2} 10^{-3} 10^{-4} 10^{-5} 10^{-6}
40 µg/ml Benzalkonium
5 µg/ml Chlorhexidine
0.15 µg/ml Norfloxacin
pTTQ18
CraA
Y42A
N45A
R124A
S135A
S162A
L163A
Y346A
L368A
M369A
10^{-1} 10^{-2} 10^{-3} 10^{-4} 10^{-5} 10^{-6}

(B)

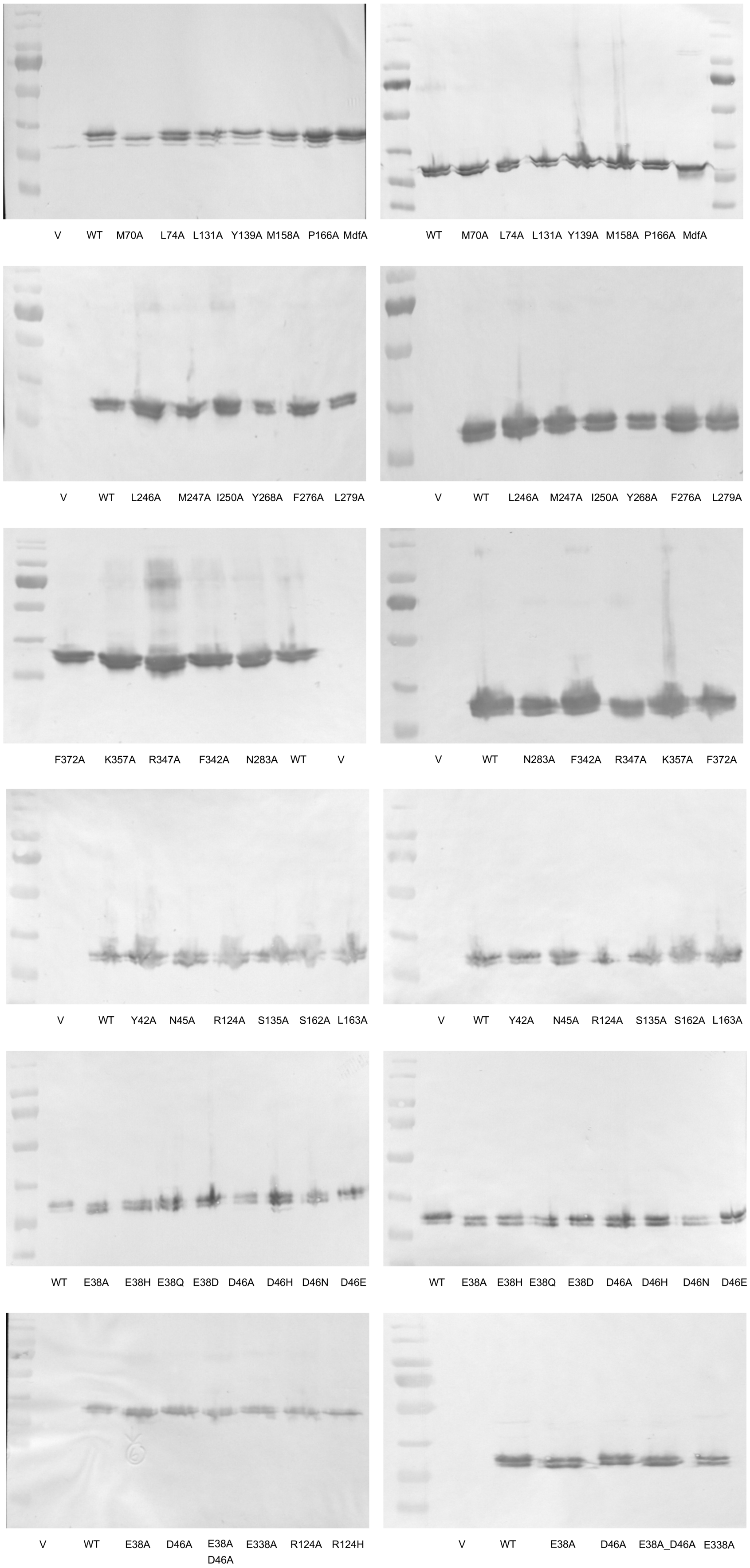
V WT M70A L74A L131A Y139A M158A P166A MdfA
WT M70A L74A L131A Y139A M158A P166A MdfA
V WT L246A M247A I250A Y268A F276A L279A
V WT L246A M247A I250A Y268A F276A L279A
F372A K357A R347A F342A N283A WT V
V WT N283A F342A R347A K357A F372A
V WT Y42A N45A R124A S135A S162A L163A
V WT Y42A N45A R124A S135A S162A L163A
WT E38A E38H E38Q E38D D46A D46H D46N D46E
WT E38A E38H E38Q E38D D46A D46H D46N D46E
V WT E38A D46A E38A D46A E338A R124A R124H
V WT E38A D46A E38A_D46A E338A

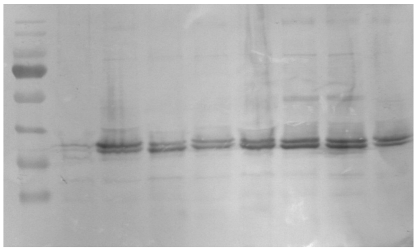

V WT E38A D46A E38A D46A E338A WT
D46A

V WT V WT V E38A D46A E38A E338A
D46A

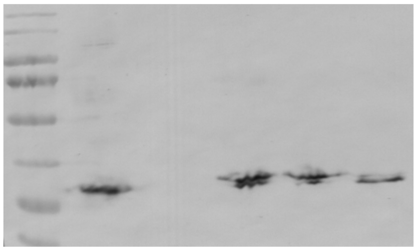

MdfA V WT Y73F Y73L

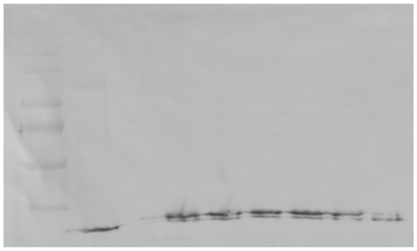

MdfA V WT Y346A L368A M369A Y73F Y73L

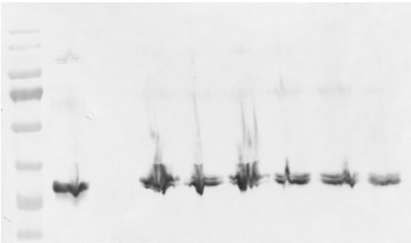

MdfA V WT Y73A Y73F Y73L R124A R124H

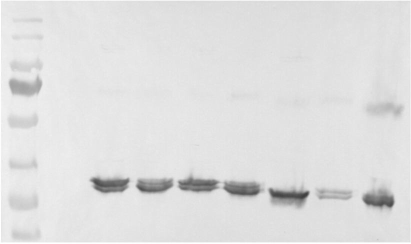

V WT Y73A Y73F Y73L MdfA E38D D46E
D46A E38A

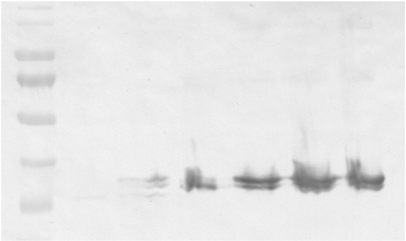

V E38D E38A L346A L368A M369A
D46A D46E

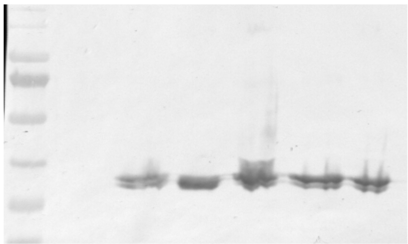

V E38D E38A L346A L368A M369A
D46A D46E

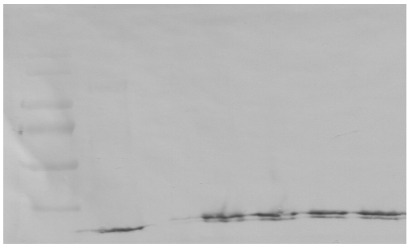

MdfA V WT L346A L348A M369A

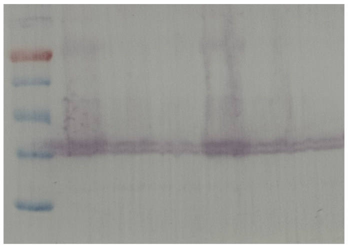

E338D E338N E338Q E338D E338N E338Q

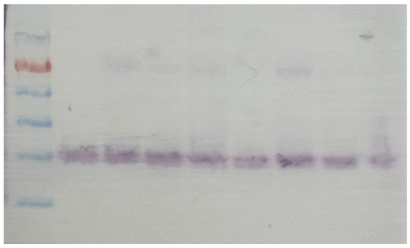

E338I Y42A Y42F Y42L E338I Y42A Y42F Y42L

FIG S4 Drug resistance profiles of *A. baumannii* WT, *A. baumannii* Δ*craA*, and *A. baumannii* Δ*craA* pBAV1K_CraA. Serially diluted overnight cultures of these strains were spotted on LB agar plates supplemented with 50 mg/L kanamycin and selected drugs (drugs and concentration given above the plate figures). Experiments were conducted at least three times and the result shown is representative.

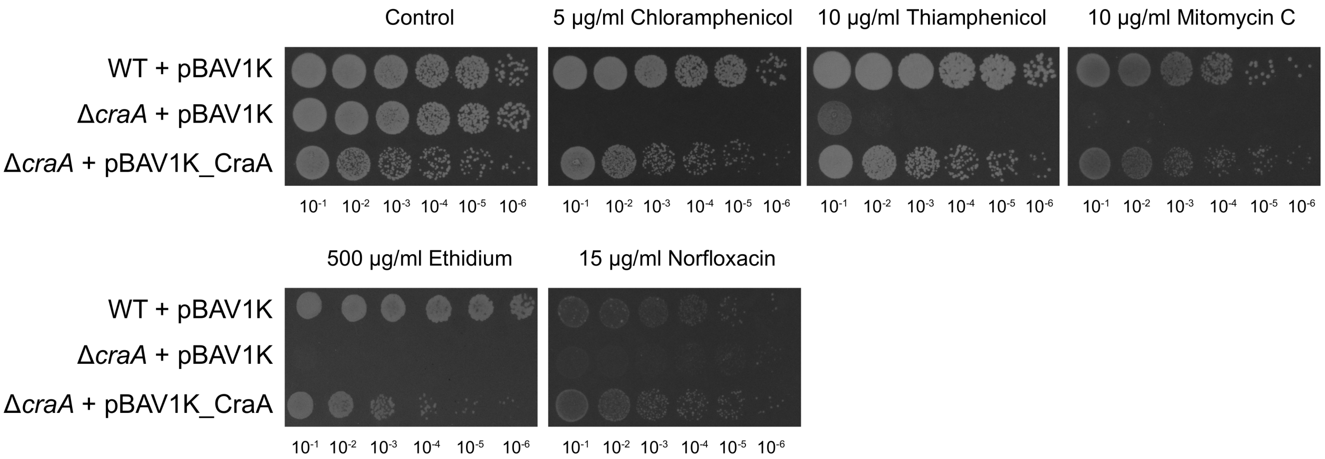

FIG S5 Substrate modes of MdfA and CraA. (A) Co-crystal structure of MdfA with chloramphenicol (carbon: black/grey; nitrogen: blue; oxygen: red; chlorine: green). Substrate Substrate docking solutions for (B) chloramphenicol (CLM); (C) ethidium (ETB); (D) mitomycin C (MIC); and (E) norfloxacin (NOR), in the CraA binding pocket (carbon: red/orange/green; nitrogen: blue; oxygen: red; chlorine: green; fluorine: cyan; sulfur: yellow). Residues shown in red (carbon): severely affected; residues shown in orange (carbon): mildly affected; residues shown in green (carbon): unaffected.

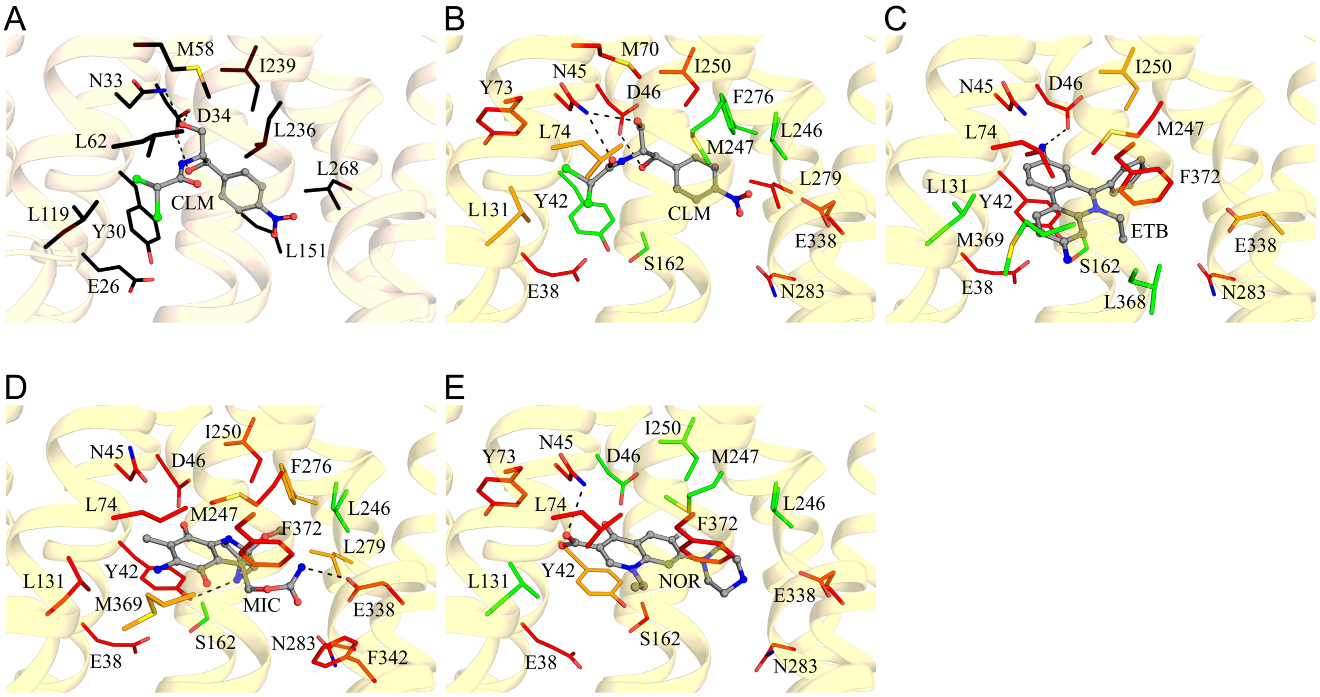

FIG S6. Drug susceptibility assays of *E. coli* BW25113 Δ*emrE*Δ*mdfA* harbouring pTTQ18 empty vector or *E. coli* cells overproducing CraA. The assays were conducted using LB agar plates supplemented with 0.2 mM IPTG, 100 µg/mL ampicillin and the indicated divalent cationic drugs with short linker (drugs and concentrations given above the plate figures). Experiments were conducted at least three times and the results shown here are representative.

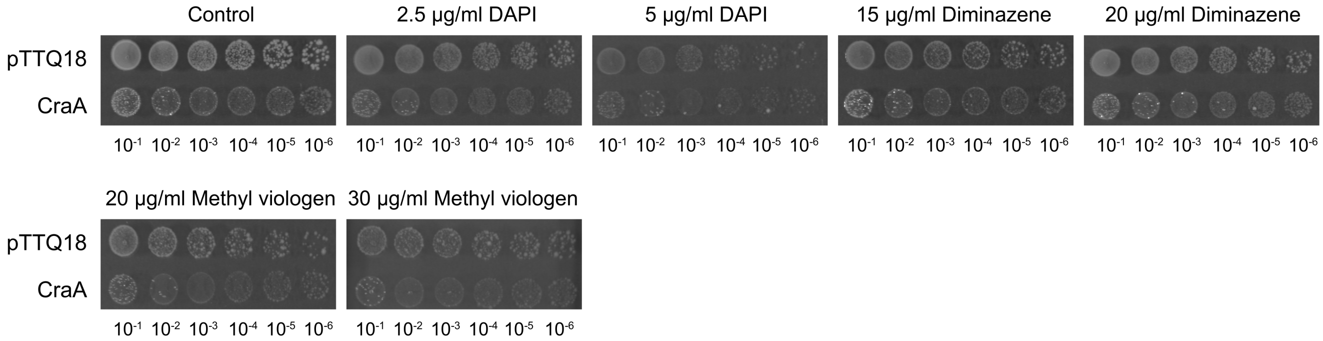

FIG S7. Interaction between R112 and Y61 in *E. coli* MdfA either in (A) inward open state (PDB: 4ZOW) (1), or (B) outward open state (PDB: 6GV1) (4). Red sphere represents water molecule, dotted line represents hydrogen bond. Residues G32, N33, Y61, T95, R112, Q115, and F174 of MdfA are homologous to G44, N45, Y73, C107, R124, Q127, and F186 of CraA.

(A) **(B)**

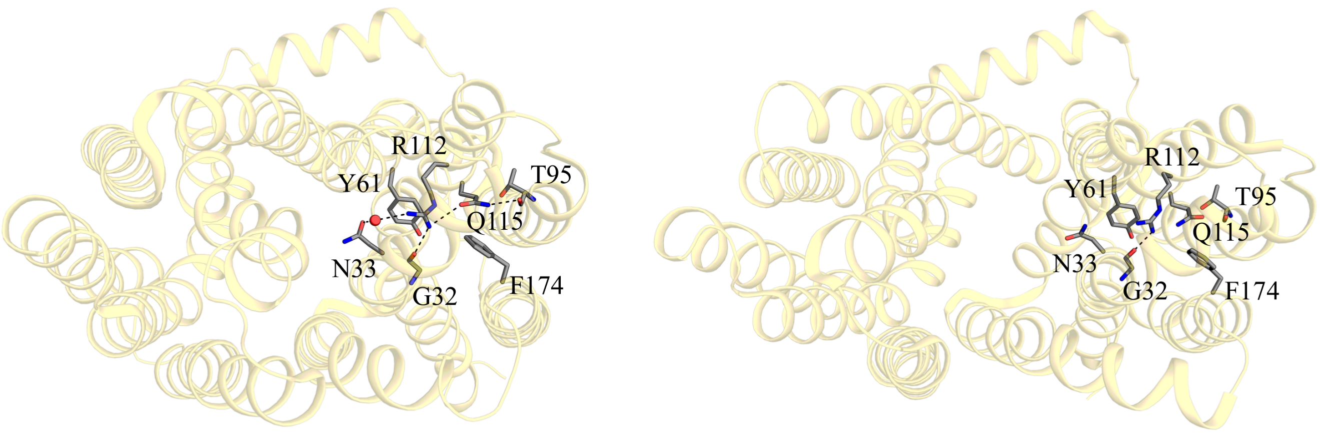

FIG S8. Residues lining the binding pocket of the homology model of CraA.

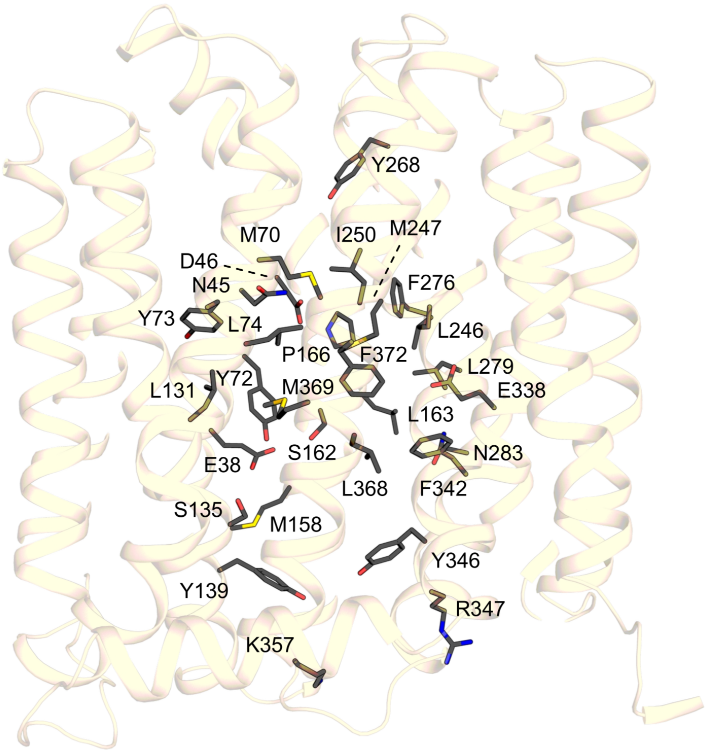

FIG S9. CraA-mediated ethidium transport assays. Active efflux of ethidium by ethidium loaded *E. coli* BW25113 Δ*emrE*Δ*mdfA* cells harboring empty vector, CraA and CraA variants were monitored fluorescently after re-energization cells with glucose, **(A)** in the absence of CCCP or **(B)** in the presence 40 µM CCCP. The ethidium bromide efflux curves were fitted by a sigmoid function with its corresponding 95% confidence interval. The ethidium bromide efflux curves for the wildtype (WT) variant and cells harboring the empty vector (Vector) in all experiments (biological and technical replicates) were fitted using a sigmoid function. As a result, the sigmoid-fitted curves for both are identical across all figures.

(A)

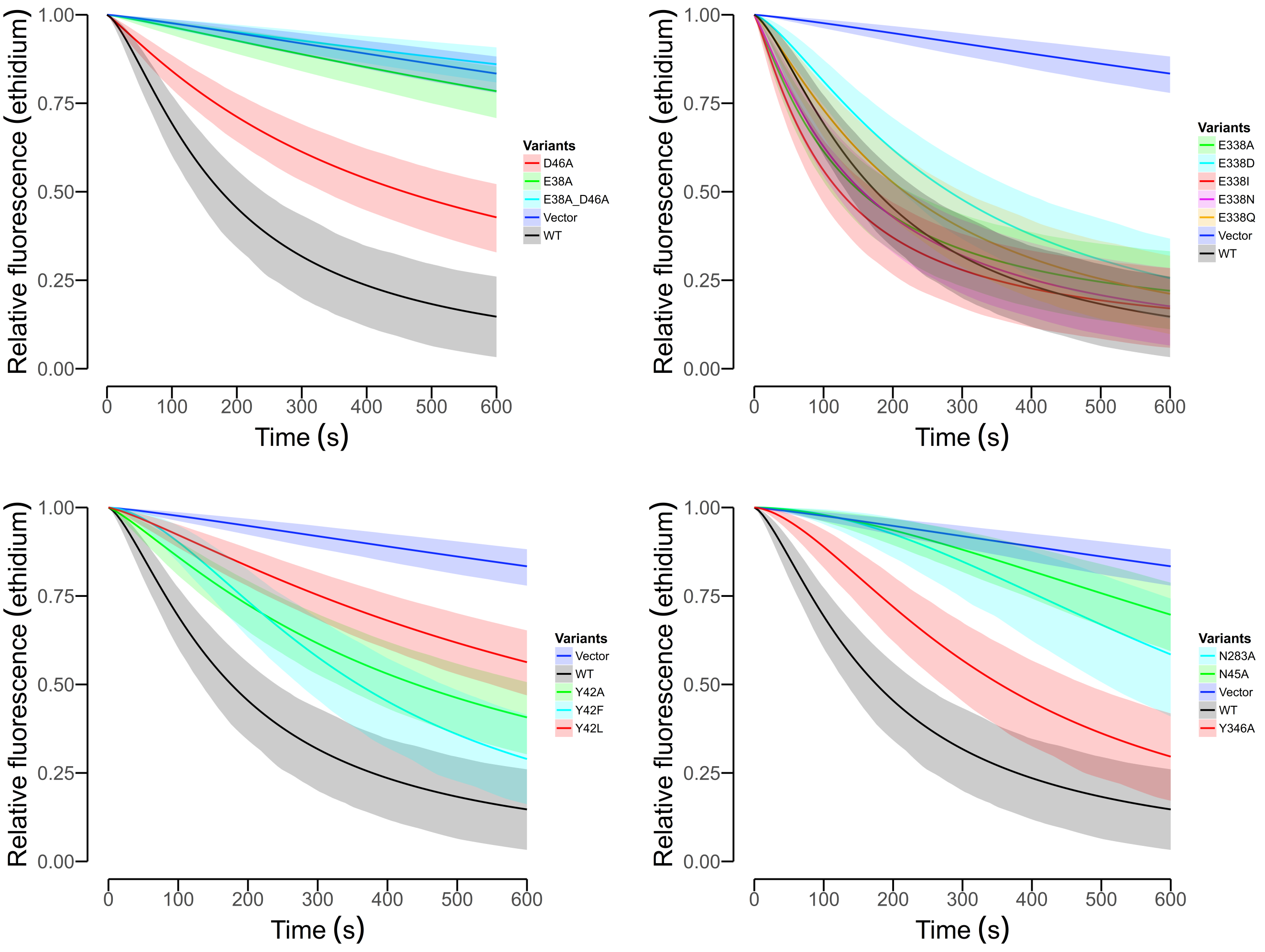

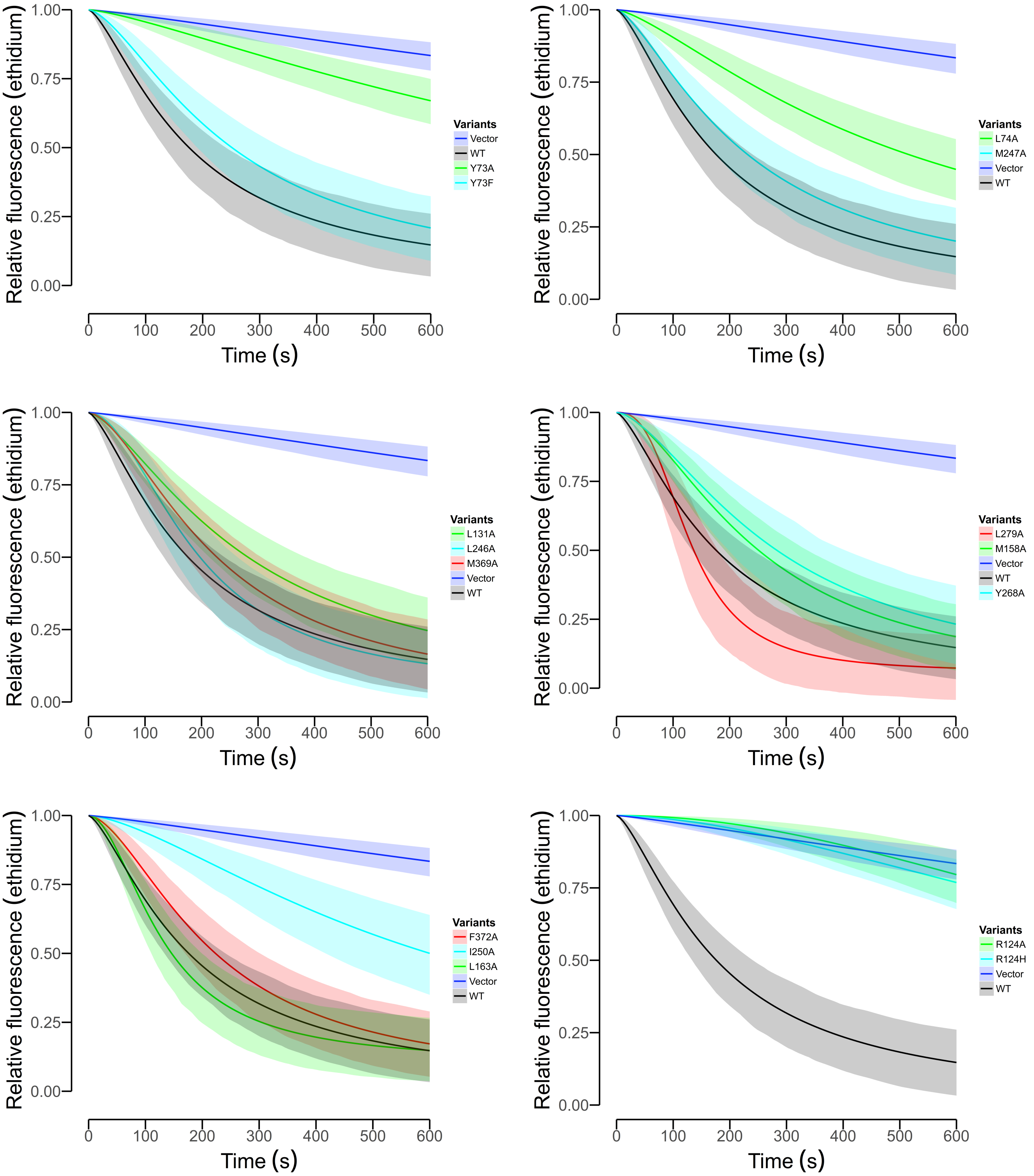
Relative fluorescence (ethidium)
Time (s)
Variants
Vector
WT
Y73A
Y73F
Variants
L74A
M247A
Vector
WT
Variants
L131A
L246A
M369A
Vector
WT
Variants
L279A
M158A
Vector
WT
Y268A
Variants
F372A
I250A
L163A
Vector
WT
Variants
R124A
R124H
Vector
WT

(B)

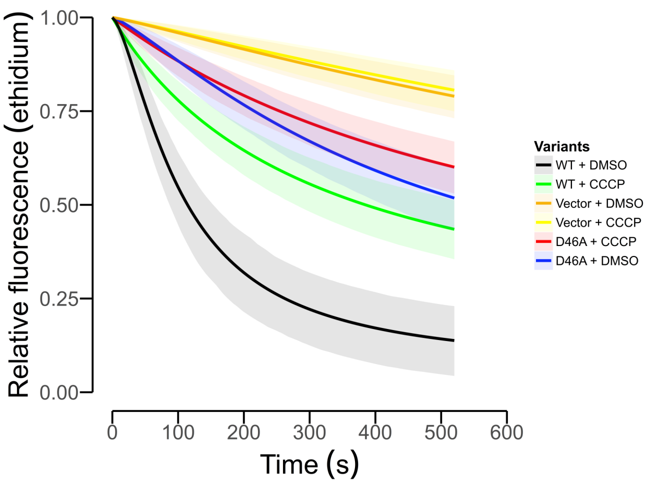

Relative fluorescence (ethidium)
1.00
0.75
0.50
0.25
0.00
0
100
200
300
400
500
600
Time (s)
Variants
WT + DMSO
WT + CCCP
Vector + DMSO
Vector + CCCP
D46A + CCCP
D46A + DMSO

FIG S10. Statistical analysis of active transport of ethidium and norfloxacin. (A) The estimates of *H* parameter calculated from fitting the ethidium bromide efflux curves by a sigmoid function. The *H* parameter is the log of the time required for the cells to remove half of the ethidium. A Type III analysis of variance using Satterthwaite's method was performed on the linear mixed effects model (see Equation 2 in Materials and Methods) to assess the effect of genotype on the *H* parameter. The analysis revealed a statistically significant difference among CraA variants, with $F(31, 274.36) = 362.9$, $p < 0.001$. (B) The estimates of relative fluorescence of accumulated norfloxacin in the *E. coli* BW25113 Δ*emrE*Δ*mdfA* cells harboring empty vector, CraA and CraA variants. A Type III analysis of variance using Satterthwaite's method was performed on the linear mixed effects model (see Equation 3 in Materials and Methods) to assess the effect of genotype on norfloxacin accumulation as measured by the fluorescence intensity. The analysis revealed a statistically significant difference among CraA variants, with $F(19, 121.26) = 87.61$, $p < 0.001$. Each dot denotes a replicate.

(A)

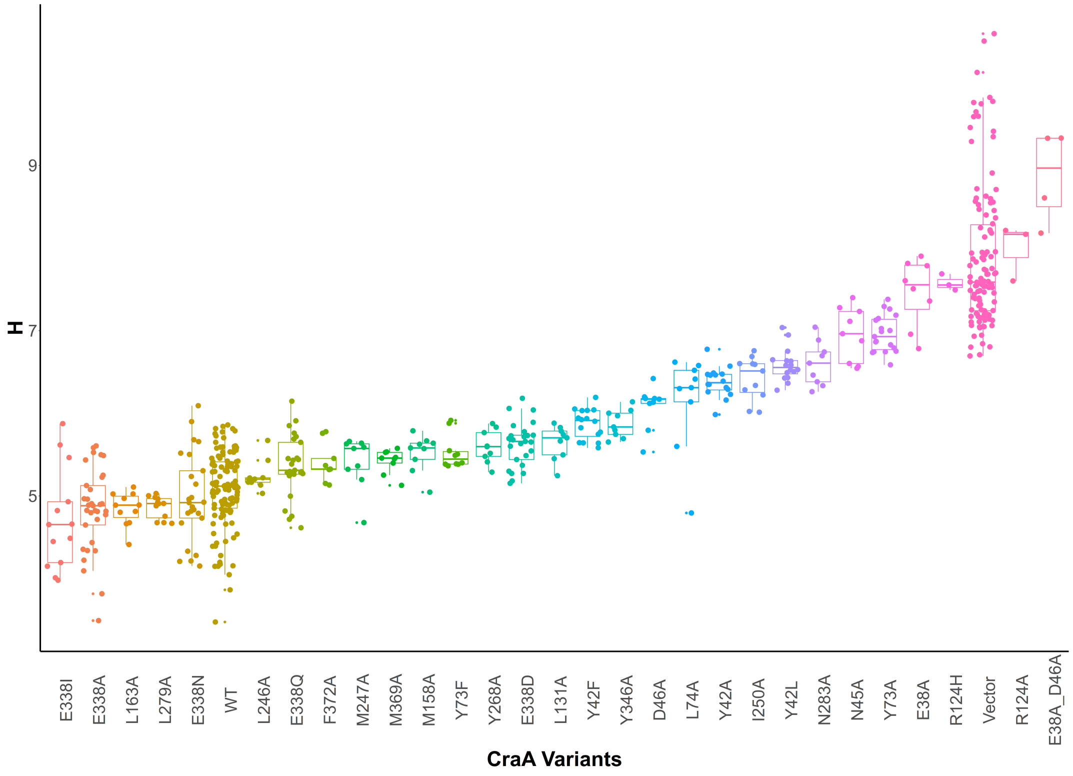

(B)

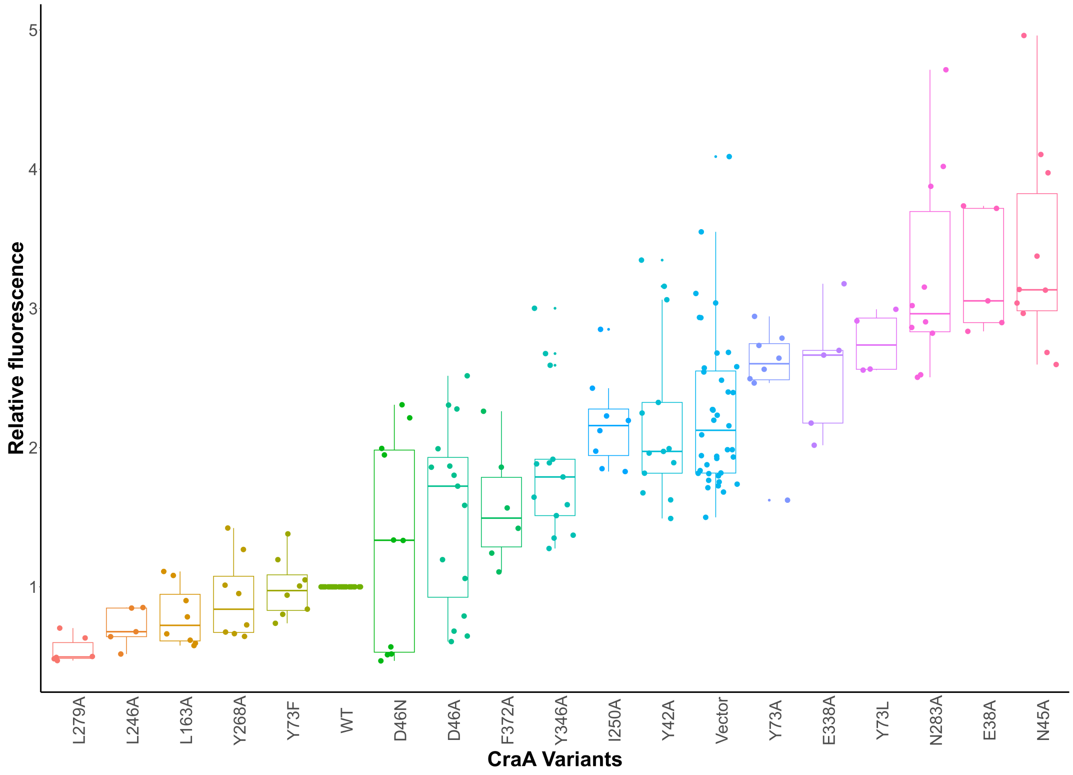
Relative fluorescence
5
4
3
2
1
L279A
L246A
L163A
Y268A
Y73F
WT
D46N
D46A
F372A
Y346A
I250A
Y42A
Vector
Y73A
E338A
Y73L
N283A
E38A
N45A
CraA Variants

FIG S11. Drug susceptibility assays of *E. coli* BW25113 Δ*emrE*Δ*mdfA* harbouring pTTQ18 empty vector, pTTQ18_craA, and CraA variants harbouring alanine substitution of membrane-embedded titratable residues. Serially diluted cells were spotted on LB agar plates supplemented with 0.2 mM IPTG, 100 mg/L ampicillin and selected drugs (drugs and concentration given above the plate figures). Experiments were conducted at least three times and the result shown is representative.

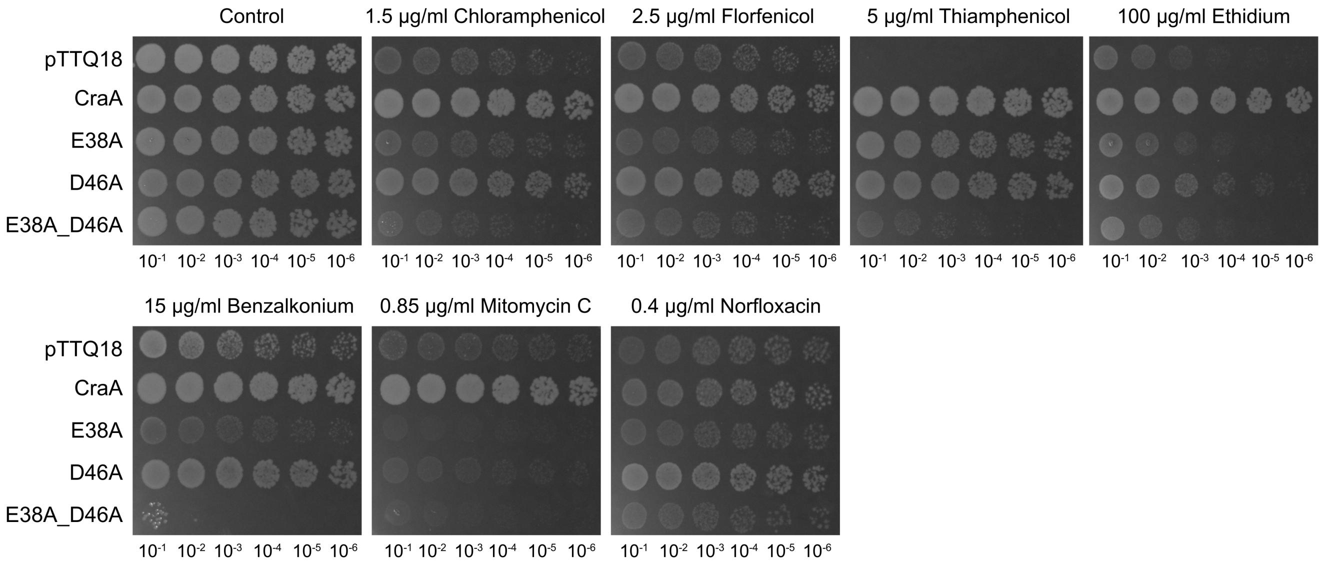

FIG S12. Gene expression analysis of *craA* in *A. baumannii* ATCC19606 in the absence or presence of chloramphenicol (Cm). The ΔC_T of the *craA* gene in the x-axis represents the calculated ΔC_T of the untreated sample (without chloramphenicol). The main effect of between “Control” and “Treatment” groups is statistically significant and large with $F(1, 6) = 78.01$, $p < 0.001$. Points and error bars represent means and standard errors, respectively, of n ≥ 3 independent experiments. The observed mean expressions of *craA* was significantly induced ($\Delta C_T = -4.7 \pm 0.3$, $\Delta\Delta C_T = 4.0 \pm 0.5$ and Tukey Honest Significant Differences test, $p < 0.001$) in response to the presence of chloramphenicol.

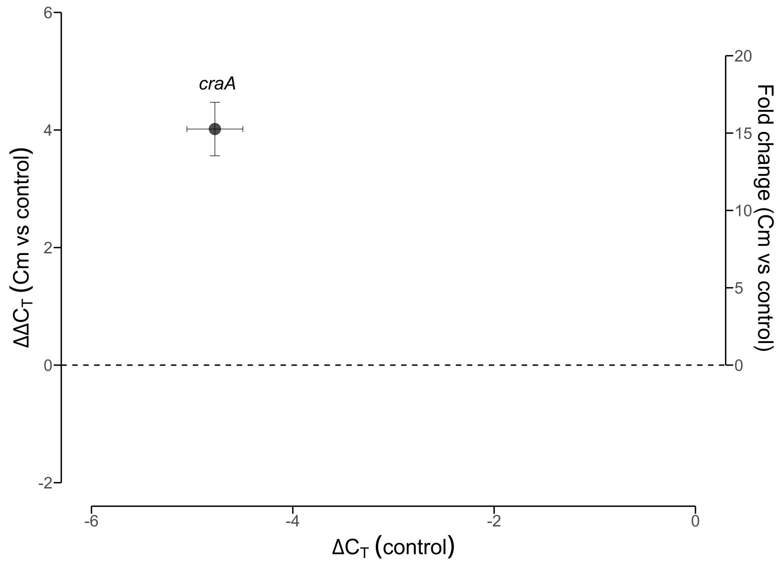

References

1. Heng J, Zhao Y, Liu M *et al.* Substrate-bound structure of the *E. coli* multidrug resistance transporter MdfA. *Cell Res* 2015; **25**: 1060 – 73.

2. Olsson MHM, Søndergaard CR, Rostkowski M, Jensen JH. PROPKA3: Consistent treatment of internal and surface residues in empirical pK_a predictions. *J Chem Theory Comput* 2011; **7**: 525 – 537.

3. Foong WE, Tam HK, Crames JJ, Averhoff B, Pos KM. The chloramphenicol/H+ antiporter CraA of *Acinetobacter baumannii* AYE reveals a broad substrate specificity, *J Antimicrob Chemother* 2019, **74**: 1192 – 201.

4. Nagarathinam K, Nakada-Nakura Y, Parthier C, Terada T, Juge N, Jaenecke F, Liu K, Hotta Y, Miyaji T, Omote H, Iwata S, Nomura N, Stubbs MT, Tanabe M. Outward open conformation of a Major Facilitator Superfamily multidrug/H+ antiporter provides insights into switching mechanism. *Nat Commun* 2018; **9**: 4005.
